# Population Genetic Structure of *Glycyrrhiza inflata* B. (Fabaceae) Is Shaped by Habitat Fragmentation, Water Resources and Biological Characteristics

**DOI:** 10.1371/journal.pone.0164129

**Published:** 2016-10-06

**Authors:** Lulu Yang, Jianjun Chen, Weiming Hu, Tianshun Yang, Yanjun Zhang, Tamura Yukiyoshi, Yanyang Zhou, Ying Wang

**Affiliations:** 1 Key Laboratory of Plant Germplasm Enhancement and Specialty Agriculture, Wuhan Botanical Garden, Chinese Academy of Sciences, Wuhan, P.R. China; 2 University of the Chinese Academy of Sciences, Beijing, P.R. China; 3 Key Laboratory of South China Agricultural Plant Molecular Analysis and Genetic Improvement, Provincial Key Laboratory of Applied Botany, South China Botanical Garden, Chinese Academy of Sciences, Guangzhou, P.R. China; 4 Maruzen Pharmaceuticals Co., LTD, Tokyo, Japan; University of Innsbruck, AUSTRIA

## Abstract

**Background:**

Habitat fragmentation, water resources and biological characteristics are important factors that shape the genetic structure and geographical distribution of desert plants. Analysis of the relationships between these factors and population genetic variation should help to determine the evolutionary potential and conservation strategies for genetic resources for desert plant populations. As a traditional Chinese herb, *Glycyrrhiza inflata* B. (Fabaceae) is restricted to the fragmented desert habitat in China and has undergone a dramatic decline due to long-term over-excavation. Determining the genetic structure of the *G*. *inflata* population and identifying a core collection could help with the development of strategies to conserve this species.

**Results:**

We investigated the genetic variation of 25 *G*. *inflata* populations based on microsatellite markers. A high level of population genetic divergence (*F*_ST_ = 0.257), population bottlenecks, reduced gene flow and moderate genetic variation (*H*_E_ = 0.383) were detected. The genetic distances between the populations significantly correlated with the geographical distances, and this suggests that habitat fragmentation has driven a special genetic structure of *G*. *inflata* in China through isolation by distance. STRUCTURE analysis showed that *G*. *inflata* populations were structured into three clusters and that the populations belonged to multiple water systems, which suggests that water resources were related to the genetic structure of *G*. *inflata*. In addition, the biological characteristics of the perennial species *G*. *inflata*, such as its long-lived seeds, asexual reproduction, and oasis ecology, may be related to its resistance to habitat fragmentation. A core collection of *G*. *inflata*, that included 57 accessions was further identified, which captured the main allelic diversity of *G*. *inflata*.

**Conclusions:**

Recent habitat fragmentation has accelerated genetic divergence. The population genetic structure of *G*. *inflata* has been shaped by habitat fragmentation, water resources and biological characteristics. This genetic information and core collection will facilitate the conservation of wild germplasm and breeding of this Chinese medicinal plant.

## Introduction

Habitat fragmentation, water resources and biological characteristics are important factors that shape the genetic structure and geographical distribution of desert plants. Analyses of the relationships between these factors and variations in population genetics should help determine the evolutionary potential and conservation strategies of genetic resources for desert plant populations [1–5]. As internal factors, biological characteristics often affect the population genetic diversity of plants [2]. Habitat fragmentation caused by nature and/or human factors is one of the main threats to biodiversity; it decreases habitat size and increases the spatial distance between habitats and populations, which results in a loss of genetic diversity and an increase in genetic differentiation [2,3,6–8]. Additionally, water resources are crucial to the evolution of desert oases. The genetic structure of desert plants could be related to river systems [9,10].

Distributed in the northwestern desert areas, including the Taklamakan Desert, the Hami basin and the Hexi Corridor of Gansu province in China, *Glycyrrhiza inflata* Batal. (Fabaceae) is one of the most important medicinal plant species that is threatened by habitat fragmentation. *G*. *inflata* has been officially recorded as the source plant of Radix Glycyrrhizae (liquorice) in Chinese pharmacopoeia [11]. The dry root and rhizomes of *G*. *inflata* have been used as Chinese medicines, flavouring agents, tobacco additives [12] and cosmetic products [13]. Liquorice is one of the oldest and most important ingredients in Chinese traditional medicine and is one of the most widely used ingredients in Chinese traditional prescriptions. For example, liquorice is an ingredient in 60% of the prescriptions included in the Treatise on Febrile Diseases and the Treatise on Spleen and Stomach [14]. Liquorice exerts many pharmacological effects, such as anti-inflammatory, hepatoprotective, anti-ulcer, anti-allergy, anti-cancer and anti-viral effects [15–20]. *G*. *inflata* flowers from May to July. The flower size variation ranges from 6 to 12 mm, with purple or light purple corolla [21]. The seeds of *G*. *inflata* are small and hard, and their germination is difficult in extremely dry and cold climates. *G*. *inflata* is a perennial herb that possesses strong underground rhizomes and reproduces both sexually by seeds and asexually by below-ground rhizomes. As a drought-tolerant and deep-rooted plant, *G*. *inflata* has a fully developed rhizome system, which is very important for sand fixing in arid and semi-arid ecosystems [14,22].

The Taklamakan Desert is the largest desert of China, and it is the largest habitat of *G*. *inflata* populations. Phylogeographical studies have found that desertification in the Tarim Basin has been present since the early Pleistocene [23]. Because of the extremely dry and cold climate, the majority of desert plants strongly depend on the water system, exemplified by their distribution along the river [9]. Plant populations distribute in scattered oases that are physically isolated by the desert. Although these natural habitats are typically patches, some plant species occur in continuous populations that are connected by river systems. Increased human activities such as agriculture, urbanization, industry and overexploitation have led to a marked decline in wild resources, aggravated desertification and increased habitat fragmentation of vegetation [3]. In addition, due to the ever-increasing demand for liquorice in recent decades, the over-excavation of liquorice has led to a marked decline in wild resources, which further accelerates the habitat fragmentation of wild *G*. *inflata* [24,25]. Although the Chinese government has prohibited the exploitation of wild liquorice plants since 2000, natural populations of *G*. *inflata* have not received effective protection due to the lack of rational use and conservation strategies. The low quality of cultivated products does not meet the requirements of Chinese pharmacopoeia, resulting in the over-excavation of wild resources. Data on the genetic diversity of *G*. *inflata* and the factors that shape this structure are essential for formulating appropriate conservation strategies and are also key factors for improving cultivated germplasms [26,27]. Previous studies on *G*. *inflata* have mainly focused on its phytochemistry and pharmacology and have paid less attention to the distribution of genetic variation across geographical ranges.

In addition, the development of core collections, which consist of subsets of an entire collection with minimal redundancy, maximizes the possible genetic diversity of a species; thus, this is one of the most effective approaches for conserving and exploring the genetic variation of a species [28,29]. At the beginning, the construction of core collections was mainly based on phenotypic data and focused on food crops, such as quinoa [30], maize [31], sorghum [32], and taro [33]. With the development of molecular markers, increasing numbers of core collections have been constructed based on genetic diversity for fruit crops or ornamental species, such as grape [34], apricot [35,36], apple [37], and litchi [38]. In addition, the maximization (M) strategy, which is based on maximizing variability, as well as nested selection methods were used to identify a subset of accessions [39–42]. The M strategy is implemented in the MSTRAT program, which determines an optimal core collection size and selects the representative individuals for a core collection of a given size [43,44]. Thus far, no core collections of *G*. *inflata* have been developed or reported for the conservation and breeding of this important medicinal plant species.

SSRs (simple sequence repeats) are comprehensively distributed throughout the plant genome and are very suitable for examining the genetic diversity of plants to identify abundant polymorphisms, co-dominant inheritance, and transferability by employing simple technology (PCR-based) [45]. With the increased availability of expressed sequence tag (EST) data, the development of EST-SSR has become more convenient and inexpensive [46]. Because EST data represent transcribed regions of the genome, EST-SSR associated with the polymorphisms of genes could be functionally more informative than genomic SSR (gSSR), although EST-SSR markers exhibit a relatively low level of polymorphisms compared with gSSR markers [47,48].

In the present study, we aimed to estimate the genetic diversity, population genetic structure patterns, and the impact of habitat fragmentation, water resources and the biological characteristics on the genetic diversity of *G*. *inflata* in China, using 20 EST-SSR markers. Furthermore, based on this genetic information, a core collection of *G*. *inflata* was identified to maximize the representativeness of the genetic diversity. Our results lay an important foundation for the conservation of wild resources and the breeding of *G*. *inflata*.

## Material and Methods

### Sampling and Field Investigation

Young leaves of 446 individuals of *G*. *inflata* were sampled from 25 natural populations, which covered almost the entire geographical range of this species in China. Leaf samples from 15 to 20 individuals were randomly sampled from each population and dried using silica gel. Because *G*. *inflata* has transverse rhizomes that are approximately 3–5 m in length, samples were collected at 5 m intervals to avoid collection from the same clone. Among these 25 populations, three populations (GJJ, GYX, GGG) were collected from the Hexi Corridor of Gansu province, four populations (XXX, SS, HJ, HS) were collected from the Hami Basin of Xinjiang province, and the remaining 18 populations (34T, TMG, KC, RQ, QM, LP, CL, MF, WL, SY, EM, 8T, 3T, SC, 48T, BC, YP, ZP) were distributed in the Tarim Basin of Xinjiang province. In addition, the spatial coordinates (latitude, longitude) of each population were recorded using a handheld GPS (Applied Magellan, Santa Clara City, CA, USA). The representative specimens of plants from each population were preserved in the herbarium of the South China Botanical Garden, Chinese Academy of Sciences (SCBG, CAS).

*G*. *inflata* was not an officially endangered or endemic plant species in 2011 (http://www.plant.csdb.cn/protectlist and http://rep.iplant.cn/protlist/2?page=42), and our study was not related to any commercial harvest of wild materials. Therefore, no specific permissions were required for collecting young leaves. Additionally, only small amounts of young leaves were collected to extract DNA in this study, and the natural conditions of wild plants were not affected. Moreover, none of the populations were located in protected areas or private land, and thus no specific permission was required to work with these populations.

### Identification of EST-SSR Loci

A total of 50,000 unigenes of *Glycyrrhiza* species were downloaded from NCBI. EST-SSR loci were identified using MISA [49], and primers were designed using Primer 3 [50]. A total of 200 EST-SSR primer pairs were designed. To select EST-SSR primer pairs with high levels of polymorphisms, eight samples from eight different populations were genotyped using the 200 EST-SSR primer pairs. The EST-SSRs that exhibited clear microsatellite peaks and high levels of polymorphism were chosen for further identification. A total of 20 EST-SSRs were selected for further study (S1 Table).

### DNA Extraction and PCR Assays

Genomic DNA was extracted from dried leaves using a modified cetyltrimethyl ammonium bromide (CTAB) method [51]. The DNA quality was tested on a 2% agarose gel, and the DNA concentration was estimated using an Eppendorf BioPhotometer (Eppendorf AG, Hamburg, Germany). All samples were diluted to 5 ng μL^-1^.

All of the samples were genotyped with 20 EST-SSRs. The PCR reaction volume was 10 μL, which contained 1 × PCR reaction buffer, 1.5 mM of MgCl_2_, 0.2 mM of dNTP mix, 0.5 μM of 5′-tagged forward primers (FAM and HEX) and 3′ general primers, 0.1 U of Taq polymerase (Thermo scientific, Wilmington, DE, USA) and 10–50 ng of template DNA. The PCR thermal profile was as follows: denaturation at 94°C for 4 min, followed by 30 cycles at 94°C for 30 s, 51–57°C for 30 s, 72°C for 45 s, and a final 10-min extension step at 72°C. The fragments were genotyped using an ABI 3730XL capillary sequencer (Applied Biosystems, Foster City, CA, USA) with LIZ-500 as an internal size standard label (Applied Biosystems). The resulting chromatograms were visualized and analysed using GeneMarker V1.5 (SoftGenetics, State College, PA).

### Effective Tests and Neutrality Tests for Microsatellite Loci

Because *G*. *inflata* can use their rhizomes for asexual reproduction, the individuals sampled from the same population could be same-clone individuals even though we sampled the plants at 5 m intervals. The same clone individuals were tested using GenAlEx 6.1 [52,53]. The putative null alleles were discovered using the software MICRO-CHECKER 2.2.3 [54]. Deviations from Hardy-Weinberg equilibrium (HWE) and linkage disequilibrium among each pair of loci in each population were tested using GENEPOP 4.0.10, with a Bonferroni correction [55,56].

Two methods for neutrality tests were employed for all of the microsatellite loci. First, LOSITAN 1.0.0 [57] software was used to detect outlier loci by evaluating the observed distribution of the *F*_ST_ values. This approach is based on the theory that the outlier loci, which are candidates for being subject to natural selection, have low or high levels of genetic differentiation compared to neutral expectations [58]. In this program, the mean neutral *F*_ST_ values were estimated for comparison with the *F*_ST_ values of all loci at the 95% confidence intervals. LOSITAN starts by running 100,000 simulations, and then the distribution of *F*_ST_ can be computed using putatively neutral loci that are derived from the simulations. Second, BayeScan v2.1 (http://cmpg.unibe.ch/software/bayescan/) was used to identified the candidate outlier loci based on the hierarchical Bayesian method [59]. The parameters were set to the default values (20 pilot runs, pilot run lengths of 5000 iterations, and a burn-in of 50,000 iterations). Then, the outliers were identified at the 90%, 95% and 99% posterior probabilities. These two methods have their own merits and drawbacks. In this study, a locus was considered an outlier if the results of one of the outlier tests was significant for this locus.

### Genetic Variation and Population Genetic Structure

Within each population, the number of effective alleles (*N*_*E*_), allelic richness (*A*_R_) and inbreeding coefficient (*F*_IS_) were analysed with FSTAT 2.9.3.2 [60,61]. The selfing rate (*s*) was estimated to be *s* = 2 *F*_IS_ / (1+ *F*_IS_) [62]. The observed (*H*_O_) and expected (*H*_E_) heterozygosities [63] were estimated by GENALEX 6.3 [52,53]. The number of different alleles in each population was calculated, and the genetic variation was measured based on the ratio of the number of different alleles in the population to the number of different alleles in the species (all 25 populations).

The genetic differentiation between populations was evaluated by the *F*_ST_ statistic, *G’*_ST_, *G”*_ST_ and *D*est, which were implemented in FSTAT2.9.3.2 and GENALEX 6.3 [51,53]. STRUCTURE 2.2 was used to detect the recent population structure and assign individuals to populations [64,65]. The number of clusters (*K*) was set from 1 to 10, and each value of *K* was determined over 10 runs with an admixture model that correlated allele frequencies using a burn-in length of 50,000 iterations and 100,000 MCMC iterations. Structure Harvester (http://taylor0.biology.ucla.edu/structureHarvester/), a web-based programme for collating the results generated by the programme STRUCTURE, was used to define the most reasonable *K* value [66]. Analysis of the molecular variance (AMOVA) was implemented in ARLEQUIN 3.0 to measure the proportions of genetic variation within and among populations [67].

### Isolation by Distance

The association between genetic distance (*F*_ST_ / (1-*F*_ST_)) and geographic distance was assessed using Mantel’s test [68] with 100,000 random permutations implemented in GENALEX 6.3 and IBD 1.52 [52,53,69].

### Detection of Population Bottlenecks

To assess each population for bottleneck signatures, we used BOTTLENECK 1.2.02 [70] software. The bottleneck was estimated using two mutation models: the infinite allele model (IAM) and the two-phased model (TPM) with 30% variance for TPM and 70% variance for SMM. The Wilcoxon signed rank test with 1,000 iterations was used. At the same time, the distribution mode of allelic frequencies shifted away from an L-shaped distribution, which also indicates recent population bottlenecks.

### Historical and Contemporary Gene Flow

Based on the coalescent [71] theory, MIGRATE 3.6.4 software [72] was used to estimate the historical gene flow *m*_h_ (*Mμ*), between pairs of populations over a long period of time (~4*N*_e_ generations) using nuclear microsatellite data under the suggested Brownian microsatellite mutation model. The ML mode was used to analyse these datasets. An adaptive heating scheme was used, and the model was run for 10 short chains with 100,000 visited and 5000 stored genealogies and two long chains with 10,000,000 visited and 50,000 sampled genealogies [73]. The contemporary gene flow was estimated by BAYESASS3-3.0.3 [74], which uses a Bayesian approach and MCMC sampling to estimate the contemporary migration rate *m*_c_ values, which represent the gene flow between pairs of populations over the past few generations. The two software systems were used to estimate the migration rates based on the neutral loci identified in outlier tests.

### Construction of Core Collections with Maximum Diversity

The M strategy (Maximization strategy) was proposed by Schoen and Brown [39] and implemented in MSTRAT 4.1 software to construct a core collection with minimum numbers of individuals in order to represent the genetic diversity of the entire collection [43]. The MSTRAT 4.1 programme can assess an optimal core collection size and choose the representative individuals [43]. In the first step, each data set was computed ten times using the random (R) and maximization (M) strategies. The results were compared and the optimal number was determined at the peak or inflection point of the curve containing all alleles of the entire collection. After determining the optimal size of the core subsets, the Shannon index (*I*) was used as the second criterion of maximization, and the core collection was constructed using 70 maximum iterations and 10 replications.

## Results

### Genetic Diversity of Wild *G*. *inflata* Populations

All 20 microsatellite loci used in this species were highly polymorphic, with the current number of alleles per locus ranging from 4 to 27. A total of 176 alleles were detected among 446 individuals. After eliminating identical clones, 397 individuals who represented 25 populations were analysed for genetic diversity. In 500 population-loci combinations, null alleles were detected in 28 combinations, which is slightly higher than the 5% level. The null alleles we detected were not concentrated in particular populations or loci. In addition, in a comparison of the *F*_ST_ values from the initial data and the revised data, we did not find any significant biases (*t*-test, *P* = 0.974). Linkage disequilibrium analyses for 20 EST-SSR markers indicated that 228 of 6,460 (3.5%) comparisons were significant after the Bonferroni correction, but they did not centre on a specific population or locus. Therefore, the linkage disequilibrium of these 20 EST-SSR markers was not significant. The test for Hardy-Weinberg equilibrium found that out of 680 locus-population combinations in *G*. *inflata*, 177, 79 and 38 (or 26.03%, 11.62% and 5.59%) showed significant deviation at *P* = 0.05, 0.01 and 0.001, respectively. Twelve populations (GJJ, GYX, SS, HJ, KC, LP, MF, EM, 8T, WL, YP, and ZP) showed significant deviations from HWE due to the excess of heterozygotes, whereas four populations (HS, QM, CL, and SC) showed significant deviations from HWE due to the deficiencies in heterozygotes. Among these 20 loci, a total of three (C1131, C142, C23) and one (C464) loci were detected as outlier loci by LOSITAN and BAYESCAN, respectively. The number of effective alleles (*N*_*E*_), allelic richness (*A*_R_), observed (*H*_O_) and expected (*H*_E_) heterozygosity, inbreeding coefficient (*F*_IS_) and selfing rate (*s*) are listed in Table 1. For all of the populations, the average of the *H*_O_ within a population was 0.460, and it ranged from 0.252 to 0.723, which was slightly higher than the *H*_E_ (averaged 0.383). The average of the inbreeding coefficient (*F*_IS_) values was -0.182 (*P* = 0.0001), and the coefficient value ranged from -0.88 to 0.107. The average number of different alleles (n_p_) within every population was 55, and the average ratio of the number of different alleles (n_p_) within one population to the number of different alleles within the species (all 25 populations) (n_s_) was 31.39%. Compared with the species level, a small number of alleles (19.89–39.20%) were present in every population, suggesting a low level of genetic variation in some populations and a high level of differentiation among the populations (Table 1). In addition, 48 private alleles were found in 18 populations but not in seven populations (GJJ, GGG, LP, MF, WL, 3T, and YP).

**Table 1:** Summary of the genetic values of 25 populations of *G*. *inflata* based on 20 SSR loci.

Population	Location	Longitude	Latitude	*N*	*N*_E_	*A*_R_	*H*_O_	*H*_E_	*F*_IS_	*s*	n_p_ (n_p_/n_s_)	Bottleneck test		
												TPM	IAM	MODE-SHIFT
GJJ	Gansu, Jinta	98.51	39.49	10	1.719	2.006	0.602	0.384	-0.474**	0	41 (23.30%)	0.00284**	0.00039**	S
GYX	Gansu, Yumen	97.12	40.17	17	1.947	2.198	0.531	0.361	-0.429**	0	49 (27.84%)	0.0166*	0.00201**	S
GGG	Gansu, Guazhou	95.45	40.30	19	1.418	1.883	0.252	0.212	0.039	0.075	52 (29.55%)	0.00516**	0.05066*	S
XXX	Xinjiang, Hami	94.47	41.54	17	2.211	2.838	0.550	0.487	-0.073	0	69 (39.20%)	0.22532	0.02299*	L
SS	Xinjiang, Shanshan	89.49	42.37	15	2.228	2.753	0.624	0.491	-0.267**	0	62 (35.23%)	0.00639**	0.00143**	S
HJ	Xinjiang, Hejing	86.20	42.18	17	2.004	2.459	0.550	0.442	-0.226**	0	54 (30.68%)	0.00714**	0.00284**	L
HS	Xinjiang, Heshuo	86.57	42.11	20	1.996	2.651	0.395	0.413	0.049^△△^	0.093	67 (38.07%)	0.38838	0.59582	L
34T	Xinjiang, Yuli	87.59	40.67	15	2.050	2.535	0.413	0.377	-0.108	0	60 (34.09%)	0.34839	0.07391	L
TMG	Xinjiang, Korla	86.12	41.47	12	2.254	2.925	0.469	0.487	0.018	0.035	68 (38.64%)	0.22875	0.02685*	L
KC	Xinjiang, Kuche	82.90	41.69	15	1.786	2.142	0.409	0.326	-0.210**	0	49 (27.84%)	0.71484	0.29578	L
RQ	Xinjiang, Ruoqiang	87.39	38.69	15	1.761	2.384	0.470	0.386	-0.169	0	57 (32.39%)	0.92171	0.2935	L
QM	Xinjiang, Qiemo	85.73	38.47	15	1.751	2.237	0.339	0.335	0.024^△^	0.047	53 (30.11%)	0.78195	0.25223	L
LP	Xinjiang, Luopu	79.93	36.98	15	2.037	2.398	0.500	0.401	-0.243**	0	56 (31.82%)	0.05768	0.01309*	L
CL	Xinjiang, Cele	80.79	36.93	16	2.078	2.429	0.397	0.411	0.008^△^	0.016	55 (31.25%)	0.00129**	0.00019*	S
MF	Xinjiang, Minfeng	82.81	37.2	15	2.030	2.376	0.468	0.393	-0.198*	0	51 (28.98%)	0.00101**	0.00015**	S
WL	Xinjiang, Yuli	86.15	41.17	21	1.883	2.365	0.426	0.404	-0.049*	0	59 (33.52%)	0.30379	0.01387*	L
SY	Xinjiang, Shaya	83.24	41.08	14	1.778	2.361	0.326	0.332	0.030	0.058	56 (31.82%)	0.4332	0.70572	L
EM	Xinjiang, Shaya	82.08	40.79	15	1.588	1.870	0.387	0.275	-0.348**	0	42 (23.86%)	0.20361	0.03418*	L
8T	Xinjiang, Alaer	80.84	40.58	16	1.487	1.606	0.447	0.256	-0.522**	0	35 (19.89%)	0.08032	0.04785*	S
3T	Xinjiang, Akesu	80.12	40.38	15	2.061	2.567	0.461	0.397	-0.080	0	63 (35.80%)	0.89057	0.41804	L
SC	Xinjiang, Shache	77.03	38.39	16	1.837	2.197	0.326	0.352	0.121^△△^	0.216	50 (28.41%)	0.25238	0.01807*	L
48T	Xinjiang, Bachu	78.18	39.39	16	2.099	2.806	0.506	0.475	-0.056	0	68 (38.64%)	0.75617	0.11399	L
BC	Xinjiang, Bachu	78.85	39.87	18	1.813	2.365	0.434	0.381	-0.062	0	60 (34.10%)	0.73809	0.56776	L
YP	Xinjiang, Qiupuhu	77.00	39.10	15	1.745	1.834	0.723	0.383	-0.786**	0	30 (21.59%)	0.00464**	0.00005**	S
ZP	Xinjiang, Zepu	77.27	38.19	18	2.077	2.631	0.498	0.420	-0.169*	0	67 (38.07%)	1	0.32473	L
MEAN				15.88	1.906	2.353	0.460	0.383	-0.167	0.0216	55 (31.39%)			

Notes: *N*, sample size; *N*_E_, mean effective allele number; *A*_R_, mean allelic richness; *H*_O_, observed heterozygosity; *H*_E_, expected heterozygosity; *F*_IS_, fixation index; */^△^, Significant at the 0.05 probability level; **/^△△^, Significant at the 0.01 probability level; *, H-W test with H1 = heterozygote excess; ^**△**^, H-W test with H1 = heterozygote deficit; s, selfing rate; n_p_, The number of different alleles within population; n_s_, The number of different alleles within species (25 populations); The significance of bottleneck effects is shown based on a two-phased model of mutation (TPM), a stepwise mutation model (SMM) and a mode shift model.

* Significant at the 0.05 probability level.

** Significant at the 0.01 probability level

### Population Structure of G. *inflata*

**Fig 1:**
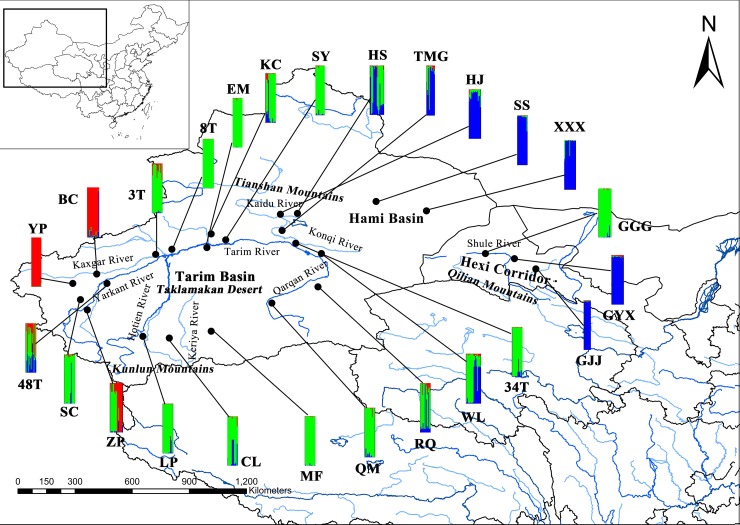
Geographic locations and genetic structure analyses of the 25 *G*. *inflata* populations in China. The 25 *G*. *inflata* populations distributed in the Hexi Corridor of Gansu province, the Hami Basin and the Tarim Basin of Xinjiang province. The spatial genetic structure assignment of wild *G*. *inflata* occurred at *K* = 3. The three colours represent three clusters that strongly matched the geographic distribution. The first cluster of populations (blue) was distributed in the Hami Basin and Hexi Corridor. The populations that were collected from the south and north rims of the Tarim Basin belonged to the second cluster (green). The third cluster populations (red) came from the west rim of the Tarim Basin.

The *F* statistics and *G* statistics tests showed that *G*. *inflata* in China had a high level of genetic differentiation (*F*_ST_ = 0.257, *G’*_ST_ = 0.244, *G”*_ST_ = 0.414 and *D*est = 0.226, *P* < 0.001). The analysis of molecular variance (AMOVA) revealed that the majority of genetic variation occurred within populations (77.06%, *P* < 0.001) and that genetic variation among populations constituted 22.94% (*P* < 0.001; Table 2). After 10 repeated runs for *K*, the Bayesian assignment with STRUCTURE showed that the best genetic structure assignment of wild *G*. *inflata* occurred at *K* = 3 (Fig 1). The three clusters strongly matched the geographic distribution. The first cluster of populations was distributed in the Hami Basin and Hexi Corridor. The populations that were collected from the south and north rims of the Tarim Basin belonged to the second cluster. The third-cluster populations came from the west rim of the Tarim Basin.

**Table 2:** Analysis of molecular variance (AMOVA) of 25 populations of *G*. *inflata*.

Source of variation	d.f.	Sum of squares	Percentage of variation	*P*
Among populations	24	901.998	22.94	< 0.001
Within populations	769	2767.219	77.06	< 0.001
Total	793	3669.217		

### IBD

**Fig 2:**
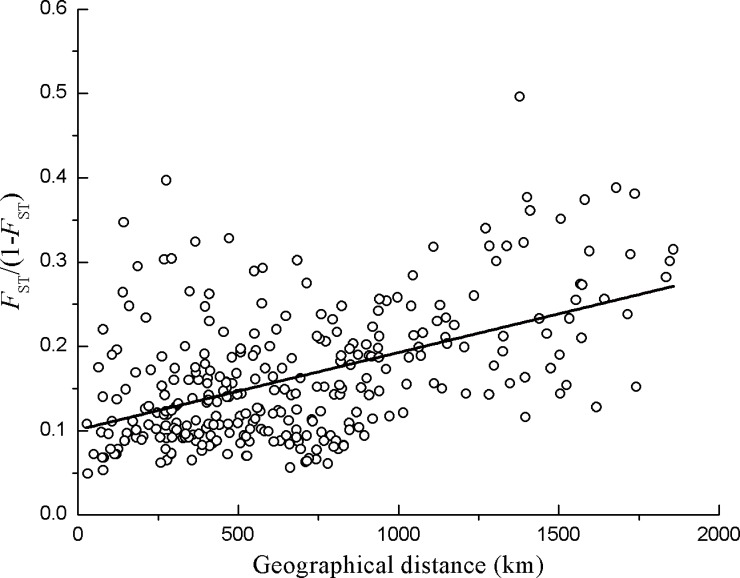
Scatter plots of the pairwise genetic distance (*F*_ST_ / (1-*F*_ST_)) versus the geographical distance (km) of all sampled populations of *G*. *inflata*. A significant positive relationship (*r* = 0.5, *P* < 0.01) was observed between the genetic distance (*F*_ST_ / (1-*F*_ST_)) and geographical distance of *G*. *inflata* populations in China.

Applying Mantel’s test showed the significant positive relationship (*r* = 0.5, *P* < 0.01) between the genetic distance (*F*_ST_ / (1-*F*_ST_)) and geographical distance of *G*. *inflata* populations in China (Fig 2).

### Population Bottlenecks

A bottleneck analysis indicated that 15 of 25 populations (GGG, GJJ, GYX, SS, HJ, CL, MF, YP, XXX, TMG, LP, WL, EM, 8T and SC) had experienced population bottlenecks under the assumption of the IPM and IAM models. In addition, eight of the 15 populations (GGG, GJJ, GYX, SS, CL, MF, 8T and YP) displayed a shifted distribution of allele frequencies (Table 1), but significant deficiency of heterozygosity was detected in only one population (GGG) in this study. Populations that have undergone population bottlenecks commonly exhibit a deficiency of heterozygosity because of inbreeding, but populations that have recently undergone a population decline exhibit an excess of heterozygosity [75]. In other words, the 14 populations underwent recent bottlenecks due to recent population declines.

### Historical and Contemporary Gene Flow

Multiple runs of BAYEASS revealed a lower contemporary gene flow among the populations of *G*. *inflata*, and the average of *m*_c_ was 0.0128, with values that ranged from 0.0078 to 0.1518 (S2 Table). MIGRATE analysis yielded a relatively high historical gene flow among the populations, and the average of *m*_h_ was 0.133, with a range from 0.031 to 0.281 (S3 Table). The historical gene flow was higher than the contemporary gene flow of *G*. *inflata* in China, which suggests that the gene flow has been decreasing as a result of habitat fragmentation.

### Construction of Core Collections with Maximum Diversity

**Fig 3:**
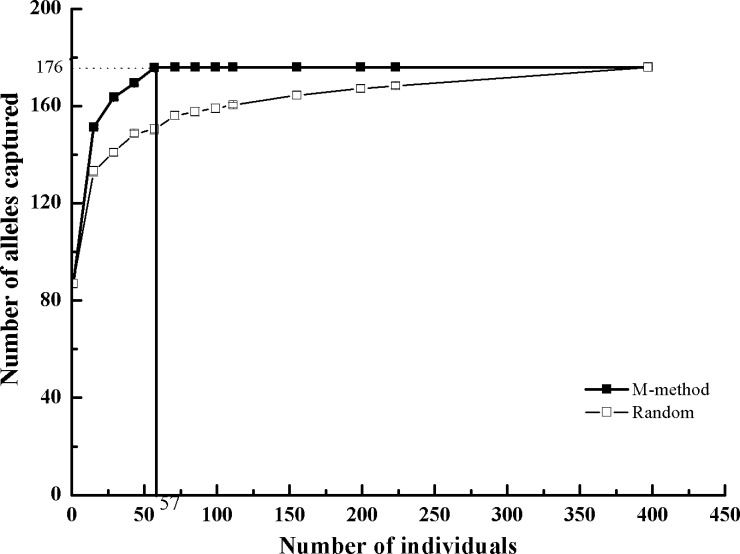
Comparison of the sampling efficiencies between the M-strategy (M-Method) and random strategy. The inflection point represents the optimal core collection size (57 accessions) that captures all 176 alleles of *G*. *inflata* in China.

Based on the M strategy, the total allelic diversity of *G*. *inflata* could be captured with 57 genotypes. Compared to a random strategy, the M strategy could capture the maximum genetic diversity with the smallest number of individuals (Fig 3). With the optimal core collection size (57 accessions) for *G*. *inflata* that captures all 176 alleles, 10 cores were obtained using the M strategy according to the Shannon index (*I*), which was used as the second criteria of maximization. The Shannon indexes (*I*) obtained from the 10 best core collections ranged from 0.395 to 0.517. Each of the 10 cores consisted of accessions from 3 clusters, which provided 17.6%, 14.5% and 7.1% of the samples to the core, respectively (Table 3, S4 Table). In addition, each core consisted of accessions from 21–24 populations, and nine populations (XXX, SS, HJ, 34T, RQ, QM, EM, SC, BC) provided more than 50% of the samples to the core (S5 Table).

**Table 3:** Genetic diversity within samples of the core collections of *G*. *inflata* classified according to the number of allele and the Shannon index (*I*).

Sample name	Alleles	I	Populations	Cluster I (blue)	Cluster II (green)	Cluster III (red)
Entire collection	176	0.656	25	108	238	51
F01 core	176	0.517	22	18 (16.7%)	35 (16.0)	4 (7.8%)
F02 core	176	0.466	21	20 (18.5%)	34 (14.3%)	3 (5.8%)
F03 core	176	0.437	21	20 (18.5%)	33 (13.9%)	4 (7.8%)
F04 core	176	0.414	24	19 (17.6%)	35 (14.7%)	3 (5.8%)
F05 core	176	0.404	24	18 (16.7%)	37 (15.5%)	2 (3.9%)
F06 core	176	0.425	23	16 (14.8%)	39 (16.4%)	4 (7.8%)
F07 core	176	0.413	22	20 (18.5%)	33 (13.9%)	4 (7.8%)
F08 core	176	0.402	22	20 (18.5%)	34 (14.3%)	3 (5.8%)
F09 core	176	0.428	22	21 (19.4%)	31 (13.0%)	5 (9.8%)
F10 core	176	0.395	21	18 (16.7%)	35 (14.7%)	4 (7.8%)
Mean	176	0.430	22.2	19 (17.6%)	34.6 (14.5%)	3.6 (7.1%)

Note: Clusters defined by Bayesian model clustering using STRUCTURE software (see Fig 1).

## Discussion

### Impact of Habitat Fragmentation on Population Genetic Diversity of *G*. *inflata*

*G*. *inflata* is distributed in the arid and semi-arid regions of northwest China. Phylogeographical studies found that desertification in the Tarim Basin has been present since the early Pleistocene [23]. A vast desert might act as a set of geographical barriers to gene exchange between populations over a long historical period. From the end of the 19th century to the beginning of the 21st century, increased human activities such as agriculture, urbanization, industry and overexploitation have led to aggravated desertification and serious habitat fragmentation after a short span of 100 years [1,76]. In our study, we found that *G*. *inflata* exhibits genetic signs of population bottlenecks, reduced gene flow, a high level of genetic differentiation between Chinese populations and low polymorphism within populations.

The significant population bottlenecks might be related to a recent decline in population size because of habitat destruction. Fourteen populations that experienced bottlenecks exhibit an excess of heterozygosity. During a population decline, the number of alleles decreases more rapidly than the heterozygosity; thus, when a population that recently underwent a population decline exhibits an excess of heterozygosity [75]. This finding is congruent with intense human activity such as agriculture, urbanization, industry and overexploitation during the past several decades, which resulted in a loss of *G*. *inflata* habitats. The southern edge of the Taklamakan desert, which comprises an extremely harsh climate and ecological environment and includes the Cele (CL), Minfeng (MF) and Luopu (LP) populations, is more fragile than the northern margin and more sensitive to human activities [77]. Agricultural development since the mid-20th century is one of the reasons underlying the fragility of this area; for example, Kaidu River (HJ and TMG), Konqi River (WL) and the area upstream of Tarim River (EM and 8T) have undergone large-scale agricultural development [78]. In addition, Qiupuhu (YP), Shache (SC), Shaya (EM), Luopu (LP), and Jinta (GJJ) are important cotton-growing bases. Urbanization and industry, such as in Hami (XXX), Shanshan (SS) and Yumen (GYX), are other reasons for the observed climate [78].

Contemporary gene flow was found to be lower than historical gene flow, which suggests that gene flow has been further reduced during the recent habitat fragmentation caused by human factors. It has been known that the gene flow of seed plants is associated with pollen or seeds [79]. The seeds of *G*. *inflata* are small and germinate with difficulty in natural habitats [80], although they can be dispersed by wind, birds and ungulates. The lower seed germination rate limits the role of seeds in gene flow. In addition, the pollinator type, quantity, behaviour and flight patterns might change, which could lead to significant pollen limitation through reduced pollen quantity or quality as fragmentation increases [81,82].

Fragmentation usually decreases the size of habitats and increases the spatial distances between habitats or populations, thus leading to ‘isolation by distance’ (IBD), which is considered to be the primary factor that leads to a high divergence between populations and a special genetic structure (SGS) [83]. Our study showed a high level of genetic differentiation (*F*_ST_ = 0.257, *G’*_ST_ = 0.244, *G”*_ST_ = 0.414 and *D*est = 0.226, *P* < 0.001) [27] that was higher than that of the rare species *Lycium ruthenicum* (*G*_ST_ = 0.2155) and lower than that of the endangered species *Gymnocarpos przewalskii* (*G*_ST_ = 0.588) [76,84] (the sympatric species with *G*. *inflata*). The Mantel test revealed that the genetic distances (*F*_ST_ / (1-*F*_ST_)) between populations were significantly correlated with the geographical distances. In addition, the populations distributed in the junctions between the clusters have mixed structure types (HS, HJ, TMG, 34T, WL, RQ, 48T and ZP) (Fig 1), and ZP, TMG, HS, HJ had higher gene flow between neighbouring populations (S6 Table and S7 Table) and thus play important roles in linking the three clusters. These findings strongly support the notion that IBD occurred among the isolated *G*. *inflata* populations. *G*. *inflata* grows in grasslands, desert riparian, floodplain meadows, roadsides and ridge. While the habitat fragmentation and distances of the populations increased, the subdivided metapopulation often followed a stepping-stone migration model in which gene flow most likely to occur between nearby populations [85]. Therefore, the gene flow between neighbouring populations tends to increase, and neighbouring populations tend to become genetically more similar. In addition, AMOVA revealed that the genetic variation among populations (22.94%) was higher than that of the rare desert species *Populus euphratica* (SSR, 5.21%) [86] and xerophyte species *Medicago ruthenica* (SSR, 10.81%) [87] and similar to that of the endangered species *Rheum tanguticum* (SSR, 21.18%) [88]. This finding also indicates that *G*. *inflata* has a high level of genetic divergence.

A low level of polymorphism within populations (n_p_/n_s_ = 31.39%) was revealed in the *G*. *inflata* species. Due to the explosive increase in the human population, the natural habitat of *G*. *inflata* has been gradually encroached upon by agricultural, industrial and urban development, resulting in a dramatic decline of the number of populations of this species. These isolated or small populations of *G*. *inflata* are more vulnerable to the impact of genetic drift, which may have caused the reduced numbers of alleles and polymorphism within the populations as well as the random fixation of differentiating alleles in different populations.

### Impact of Water Resources on the Population Structure of *G*. *inflata*

The effects of population fragmentation might be counteracted by the water system [3,89]. Because the desert ecosystem is characterized by low annual precipitation and high evaporation, water for desert plants is more important than for plants in other ecosystems. Under the extremely arid desert climate of the Tarim Basin, large oases form due to rivers that are fed by meltwater from the snow and ice in the adjacent mountains [9,90]. Although most of the rivers are seasonal or ephemeral, sparse but continuous vegetation cover near the rivers sustains a chain of oases along the desert periphery [91]. In this study, all of the populations of *G*. *inflata* were structured into three clusters and belong to multiple water systems. The western populations belong to the Kaxgar River water system, which originates in Pamirs. The northern populations belong to the Tarim River, Kaidu River and Konqi River water system, which originates from the Tianshan Mountains. The southern populations belong to the Ho-tien River, Yarkant River and Qarqan River water system, which originate from the Kunlun Mountains. The eastern populations belong to the Shule River water system, which originates from the Qilian mountains. It was also found that the population genetic structure corresponded well to the water systems and different population clusters belonging to different water systems. The genetic differentiation could be due to the origination of the water systems. In addition, there are 12 populations that are distributed in the south and north rims of the Tarim Basin that were isolated by the Taklamakan Desert, but they were clustered together in the structure assignment tests. The Ho-tien River and Keriya River might play important roles in linking these isolated populations and promoting historical gene flow between the northern and southern populations. Although the two rivers have been drying up year round or seasonally, they used to flow through the Taklamakan Desert and reached the Tarim River of the northern fringe of the Tarim Basin during several different geological times [92,93].

In addition, the absence of water might be one of the reasons underlying the aggravation of plant population fragmentation. With 20–70 mm of annual precipitation in the Taklamakan Desert, plants can grow only if they have access to groundwater, lakes or rivers [10]. However, now this water is captured in canal systems and used for the irrigation of arable fields (e.g., cotton, wheat, corn). Since the beginning of the twentieth century, especially since the 1950s, the expansion of irrigation caused a reduction in the flow of the lower reaches of the rivers, which resulted in the death of downstream vegetation and oases desertification. For example, Bruelheide found a trend towards a decreasing width of the indigenous vegetation belt that resulted from the advancing desert and the expansion of arable land in the Qira oasis over the past 50 years. Although humans have survived in the oasis for approximately 2000 years, the effects of human populations on the oases was quite small before the appearance of extensive irrigation systems [9].

### Biological Characteristics Impact the Genetic Variation of *G*. *inflata*

Here, the *G*. *inflata* populations still maintain moderate genetic variation (*H*_E_ = 0.383). When genomic and EST-SSRs were compared using the same set of genotypes, the number of polymorphisms detected with EST-SSRs was lower than that of genomic SSRs [94,95]. Simko also found that the average marker heterozygosity (*H*_E_ = 0.32) was significantly reduced in the expressed sequence tag EST-SSRs compared with anonymous SSRs (*H*_E_ = 0.59) in lettuce [96]. Nevo and Beiles [97] have proposed that organisms that inhabit arid and semi-arid zones should exhibit greater levels of genetic variability at the population level compared with those that inhabit other habitats. Wang also found that *Populus euphratica* has a higher genetic variation (SSR, *H*_E_ = 0.713–0.878) [86]. By comparison, we found that the temporary maintenance of the moderate genetic viability of *G*. *inflata* may have be due to its biological characteristics as a desert plant. First, as a perennial species, *G*. *inflata* may be expected to be less vulnerable than annual species to habitat fragmentation [2]. The above-ground parts of *G*. *inflata* (the aerial portion) are annual, but the underground parts are perennial [21]. Although the aerial portion of *G*. *inflata* plants die in the winter, these parts turn green again during the following spring. Compared to short-lived species, individual plants or sub-populations of long-lived species such as *G*. *inflata* are more likely to persist, easing the pressure from habitat fragmentation. Second, asexual reproduction using underground roots or rhizomes can produce individuals that are genetically identical to their parents, which is an effective mechanism for the protection of genetic diversity. In desert plants, asexual reproduction is one of the main adaptive traits [2]. Third, the small, hard seeds of *G*. *inflata* germinate with difficulty in extremely dry and cold climates [80,98]. However, due to the hard seed coat, the seeds of *G*. *inflata* can remain active during long periods of dormancy. Seeds that fall into the soil can form a seed bank and germinate gradually according to the degree of damage to the seed coat. The seed bank provides the plants with the opportunity to re-establish a population following destruction. Fourth, the Taklamakan Desert shows typical oasis ecology in which oases are distributed near the rivers. The desert provides a natural buffer zone between the oases. In other words, the distribution of *G*. *inflata* in the desert may have already experienced habitat fragmentation for a substantial amount of time. Thus, *G*. *inflata* may have acquired adaptability and resistance to the habitat fragmentation due to the removal of maladapted genotype or biological characteristics. For example, some researchers have found that, compared to *Glycyrrhiza uralensis* Fisch, which is distributed in the prairie, there are more adventitious buds on the underground stems of *G*. *inflata* and marginal, somewhat undulate *G*. *inflata* leaflets, which are beneficial for the survival and expansion of the *G*. *inflata* populations in the desert [11,21].

### A Core Collection Was Developed that Captures the Allelic Diversity of *G*. *inflata*

Maintaining genetic diversity is critical for the long-term survival of a species because a loss of variation might dramatically limit the adaptability of populations that are responding to changing environments [76]. The purpose of core collections is to facilitate the protection and use of genetic variation by providing a set of accessions that exhibit the genetic diversity that is available in a larger collection [29]. In the present study, a core collection of *G*. *inflata* with 57 accessions was identified, and this collection successfully covered the allelic diversity of the *G*. *inflata* species. This collection is the first core collection of *Glycyrrhiza* Linn. The core collection of *G*. *inflata* that was developed will facilitate the breeding of new cultivars of *G*. *inflata* to meet market demand as well as meeting the demands of genetic and phytochemical studies. In addition, we observed that nine populations (XXX, SS, HJ, 34T, RQ, QM, EM, SC, BC) provided more than 50% of the samples to the core (S5 Table). Among these, XXX, SS, BC, WL and RQ are the populations that contribute the most diversity to the core collection. Thus we believe that the localities of Hami, Shanshan, Yuli, Ruoqiang and Bachu should be prioritized in future conservation plans.

## Conclusions

As expected, the rare species *G*. *inflata* possessed a high level of genetic divergence and experienced population bottlenecks in small populations first due to habitat fragmentation that was caused by nature and human factors. Limited gene flow combined with increasingly fragmented habitats has driven the special existing genetic structure of *G*. *inflata* in China. However, the water system played an important role in countering the effects of isolation. Because of the biological characteristics of this perennial species, such as its asexual propagation, long-lived seeds, and ecological adaptability to oases, *G*. *inflata* can temporarily maintain a moderate level of genetic variability. Based on the genetic information we obtained, a core collection of *G*. *inflata* that included 57 accessions was further identified, which captured the main allelic diversity. Our results will play an important role in developing conservation strategies for the sustainable utilization of wild resources as well as for the breeding and cultivation of *G*. *inflata*.

## Supporting Information

S1 Table The 20 pairs of EST-SSR primers. (DOC)

S2 Table Contemporary migration rates between 25 populations of *G*. *inflata* in China. (DOC)

S3 Table Historical migration rates between 25 populations of *G*. *inflata* in China. (DOC)

S4 Table The sample IDs of the ten best core collections. (DOC)

S5 Table The number of core individuals in every population. (DOC)

S6 Table The historical and contemporary migration rates of LP and neighbour populations from two clusters. (DOC)

S7 Table The historical and contemporary migration rates of HJ, HS, TMG and neighbour populations from two clusters. (DOC)
