# Correction: Population Genetic Structure of *Glycyrrhiza inflata* B. (Fabaceae) Is Shaped by Habitat Fragmentation, Water Resources and Biological Characteristics

**DOI:** 10.1371/journal.pone.0170867

**Published:** 2017-01-24

**Authors:** Lulu Yang, Jianjun Chen, Weiming Hu, Tianshun Yang, Yanjun Zhang, Tamura Yukiyoshi, Yanyang Zhou, Ying Wang

There is an error in affiliation 4 for the authors Tamura Yukiyoshi and Yanyang Zhou. Affiliation 4 should be: Maruzen Pharmaceuticals Co., LTD, Hiroshima, Japan.
