# Supplementary material for: Population Genetic Structure of Glycyrrhiza inflata B. (Fabaceae) Is Shaped by Habitat Fragmentation, Water Resources and Biological Characteristics
Source: PLoS One. 2016 Oct 6;11(10):e0164129. doi: 10.1371/journal.pone.0164129 (PMC5053598; doi:10.1371/journal.pone.0164129)
Supplement: S1 Table — (DOC) [file pone.0164129.s001.doc]

**S1 Table. The 20 pairs of EST-SSR primers**

| **ID** | **SSR type** | **Forward primer** | **Reverse primer** | **Product size (bp)** |
| --- | --- | --- | --- | --- |
| **C1131** | (TCTTGC)4 | CCTGCTGATCTCAGTCTCGC | AACCCCAAAACCACTACACG | 118 |
| **C552** | (AAC)8 | CCTCAACACTGAACGCGTAA | GCTTGTCGCTCTTCGATTTC | 213 |
| **C1452** | (GT)9 | GTTAATGCGATTTATCCGGG | GGCAGATCTTCCAAATTCCA | 126 |
| **C1213** | (CTGT)4 | TAGGATACGACCCAACCTGC | CGGAATCCTCAACTCCAAAA | 204 |
| **C967** | (AGAT)6 | TGCTGTGCTCACACTCAGAA | GTGTCGTAGCCGAAGAGGAG | 228 |
| **C1041** | (AGA)6 | AGGAAGCCAAACAGTGGCTA | CGAACCACCTGATGATCCTT | 162 |
| **C23** | (CCAATT)4 | TCTCGCCACTGTTTCTGATG | AAAACAACCCATTTGGGACA | 137 |
| **C1329** | (CTT)6 | ACACCCCAAACACGTTCAAT | GATGCATTGGAGAAGCACAA | 215 |
| **C48** | (CACT)4 | AAACTCCGAATGAGTAGTCCGA | TCGACATCATCGAACAGAGC | 140 |
| **C1379** | (CAT)6 | CCTCAGGCCTCAACCTAACC | GTTACCACCTCGATCTCCGA | 179 |
| **C282** | (ATA)6 | GATTCACCATCAAGTCAATCACA | TCCTCAAGGGTGATACCCAG | 262 |
| **C106** | (TATG)4 | TTTGGTGCCATTGCTATTGA | GCACCATACATTGCGATTCA | 256 |
| **C861** | (TCT)7 | CACCCCGAAATTCTGAAAGA | AATTGGAGTGGAAATGCTGG | 190 |
| **C1029** | (ATC)8 | CCTTGACGTGGGACTTGTCT | ATCGTTAACCCCCAACATCA | 279 |
| **C1087** | (GT)14 | ATAGGCGTCCTCTCTATGCG | CCGAGTCCAATCTCAATGATG | 166 |
| **C1278** | (GAA)14 | CCTTCTTCCCATTTTCACCA | ACCCACCACTTTGTACCCAA | 274 |
| **C895** | (AGTG)4 | TGCTGTTTGGAAAAGGAAAAA | TACCATTCCTTCGCTTGGAG | 187 |
| **C464** | (AGC)6 | CGGTGGAAAACAAAAACACA | GGTGAGTTGACTCGTTGGGT | 253 |
| **C1404** | (ATAG)4 | GGGGAGTTAAAGGGGAACAA | GCATTCCAAGAGGAACAAGC | 194 |
| **C145** | (AC)13 | CCACATGCTCACGAGCTTTA | CTCCAGTGCTTCCCTTTCTG | 280 |
