# Supplementary material for: Population Genetic Structure of Glycyrrhiza inflata B. (Fabaceae) Is Shaped by Habitat Fragmentation, Water Resources and Biological Characteristics
Source: PLoS One. 2016 Oct 6;11(10):e0164129. doi: 10.1371/journal.pone.0164129 (PMC5053598; doi:10.1371/journal.pone.0164129)
Supplement: S2 Table — (DOC) [file pone.0164129.s002.doc]

**S2 Table.** Contemporary migration rates between 25 populations of *G.inflata* in China.

|  | GJJ | GYX | GGG | XXX | SS | HJ | HS | 34T | TMG | KC | RQ | QM | LP | CL | MF | WL | SY | EM | 8T | 3T | SC | 48T | BC | YP | ZP |
| --- | --- | --- | --- | --- | --- | --- | --- | --- | --- | --- | --- | --- | --- | --- | --- | --- | --- | --- | --- | --- | --- | --- | --- | --- | --- |
| GJJ |  | 0.0062 | 0.0064 | 0.0076 | 0.0078 | 0.0073 | 0.0073 | 0.0077 | 0.0067 | 0.0068 | 0.0071 | 0.0077 | 0.0072 | 0.0083 | 0.0075 | 0.0075 | 0.0075 | 0.0082 | 0.0077 | 0.0073 | 0.0084 | 0.0079 | 0.0078 | 0.0060 | 0.0073 |
| GYX | 0.0051 |  | 0.0029 | 0.0042 | 0.0049 | 0.0051 | 0.0045 | 0.0043 | 0.0045 | 0.0044 | 0.0048 | 0.0038 | 0.0437 | 0.0061 | 0.0041 | 0.0040 | 0.0053 | 0.0053 | 0.0050 | 0.0051 | 0.0046 | 0.0045 | 0.0041 | 0.0035 | 0.0050 |
| GGG | 0.006 | 0.0057 |  | 0.0065 | 0.0052 | 0.0066 | 0.0049 | 0.0048 | 0.0106 | 0.0063 | 0.0068 | 0.0060 | 0.0061 | 0.0055 | 0.0054 | 0.0058 | 0.0283 | 0.0053 | 0.0049 | 0.0063 | 0.0060 | 0.0054 | 0.0066 | 0.0060 | 0.0056 |
| XXX | 0.0063 | 0.0065 | 0.0073 |  | 0.0099 | 0.0063 | 0.0062 | 0.0054 | 0.0537 | 0.0066 | 0.0072 | 0.0238 | 0.0071 | 0.0067 | 0.0056 | 0.0058 | 0.0070 | 0.0061 | 0.0062 | 0.0064 | 0.0067 | 0.0069 | 0.0062 | 0.0068 | 0.0061 |
| SS | 0.0079 | 0.0070 | 0.0081 | 0.0077 |  | 0.0074 | 0.0079 | 0.0075 | 0.0076 | 0.0079 | 0.0073 | 0.0084 | 0.0085 | 0.0078 | 0.0076 | 0.0079 | 0.0084 | 0.0084 | 0.0083 | 0.0072 | 0.0087 | 0.0083 | 0.0078 | 0.0082 | 0.0073 |
| HJ | 0.0072 | 0.0079 | 0.0078 | 0.0078 | 0.0079 |  | 0.0073 | 0.0075 | 0.0740 | 0.0071 | 0.0075 | 0.0075 | 0.0078 | 0.0079 | 0.0076 | 0.0078 | 0.0079 | 0.0072 | 0.0076 | 0.0075 | 0.0075 | 0.0077 | 0.0076 | 0.0081 | 0.0078 |
| HS | 0.0061 | 0.0069 | 0.0073 | 0.0068 | 0.0071 | 0.0071 |  | 0.0076 | 0.0489 | 0.0073 | 0.0072 | 0.0069 | 0.0072 | 0.0077 | 0.0073 | 0.0074 | 0.0106 | 0.0072 | 0.0076 | 0.0071 | 0.0071 | 0.0072 | 0.0078 | 0.0074 | 0.0075 |
| 34T | 0.0073 | 0.0059 | 0.0069 | 0.0061 | 0.0063 | 0.0067 | 0.006 |  | 0.0099 | 0.0064 | 0.0065 | 0.0126 | 0.0073 | 0.0199 | 0.0063 | 0.0064 | 0.0257 | 0.0061 | 0.0065 | 0.0065 | 0.0064 | 0.0067 | 0.0075 | 0.0071 | 0.0067 |
| TMG | 0.0076 | 0.0075 | 0.0066 | 0.0070 | 0.0069 | 0.0065 | 0.0072 | 0.0071 |  | 0.0087 | 0.0108 | 0.0068 | 0.0080 | 0.0097 | 0.0067 | 0.0079 | 0.0081 | 0.0077 | 0.0099 | 0.0064 | 0.0068 | 0.0074 | 0.0068 | 0.0062 | 0.0074 |
| KC | 0.0062 | 0.0061 | 0.0057 | 0.0065 | 0.0069 | 0.0069 | 0.0071 | 0.0061 | 0.0121 |  | 0.0061 | 0.0064 | 0.0069 | 0.0061 | 0.0072 | 0.0063 | 0.0099 | 0.0073 | 0.0081 | 0.0067 | 0.0071 | 0.0068 | 0.0068 | 0.0062 | 0.0067 |
| RQ | 0.0066 | 0.0065 | 0.0068 | 0.0075 | 0.0069 | 0.0064 | 0.0065 | 0.0067 | 0.0215 | 0.0075 |  | 0.0078 | 0.0075 | 0.0091 | 0.0066 | 0.0064 | 0.0136 | 0.0064 | 0.0067 | 0.0073 | 0.0072 | 0.0073 | 0.0072 | 0.0060 | 0.0081 |
| QM | 0.0070 | 0.0053 | 0.0070 | 0.0080 | 0.0074 | 0.0067 | 0.0069 | 0.0051 | 0.0068 | 0.0068 | 0.007 |  | 0.0062 | 0.0070 | 0.0074 | 0.0064 | 0.0076 | 0.0059 | 0.0059 | 0.0067 | 0.0063 | 0.0069 | 0.0067 | 0.0056 | 0.0066 |
| LP | 0.0059 | 0.0065 | 0.0066 | 0.0067 | 0.0067 | 0.0067 | 0.0070 | 0.0068 | 0.0073 | 0.0073 | 0.0073 | 0.0059 |  | 0.0239 | 0.0076 | 0.0063 | 0.0084 | 0.0068 | 0.0072 | 0.0067 | 0.0063 | 0.0075 | 0.0066 | 0.0066 | 0.0054 |
| CL | 0.0070 | 0.0060 | 0.0059 | 0.0073 | 0.0067 | 0.0066 | 0.0068 | 0.0100 | 0.0076 | 0.0188 | 0.0062 | 0.0064 | 0.0060 |  | 0.0071 | 0.0064 | 0.0116 | 0.0070 | 0.0397 | 0.0062 | 0.0063 | 0.0062 | 0.0062 | 0.0066 | 0.0073 |
| MF | 0.0067 | 0.0079 | 0.0073 | 0.0064 | 0.0075 | 0.0066 | 0.0068 | 0.0062 | 0.0063 | 0.0068 | 0.0072 | 0.0062 | 0.0076 | 0.0062 |  | 0.0064 | 0.0095 | 0.0066 | 0.0054 | 0.0062 | 0.0073 | 0.0053 | 0.0068 | 0.0058 | 0.0059 |
| WL | 0.0071 | 0.0072 | 0.0072 | 0.0073 | 0.0072 | 0.0070 | 0.0075 | 0.0074 | 0.0145 | 0.0078 | 0.0070 | 0.0072 | 0.0073 | 0.0079 | 0.0086 |  | 0.0674 | 0.0075 | 0.0070 | 0.0068 | 0.0073 | 0.0070 | 0.0069 | 0.0068 | 0.0071 |
| SY | 0.0074 | 0.0070 | 0.0072 | 0.0063 | 0.0064 | 0.0073 | 0.0065 | 0.0067 | 0.0065 | 0.0067 | 0.0061 | 0.0065 | 0.0089 | 0.0080 | 0.0072 | 0.0059 |  | 0.0081 | 0.0090 | 0.0070 | 0.0067 | 0.0073 | 0.0066 | 0.0067 | 0.0062 |
| EM | 0.0056 | 0.0074 | 0.0068 | 0.0079 | 0.0067 | 0.0064 | 0.0064 | 0.0063 | 0.0066 | 0.0065 | 0.0074 | 0.0055 | 0.0068 | 0.0072 | 0.0064 | 0.0068 | 0.0089 |  | 0.0090 | 0.0069 | 0.0062 | 0.0070 | 0.0065 | 0.0068 | 0.0065 |
| 8T | 0.0057 | 0.0067 | 0.0062 | 0.0070 | 0.0066 | 0.0058 | 0.0068 | 0.0062 | 0.0066 | 0.0065 | 0.0056 | 0.0061 | 0.0051 | 0.0055 | 0.0067 | 0.0074 | 0.0062 | 0.0054 |  | 0.0062 | 0.0071 | 0.0060 | 0.0071 | 0.0069 | 0.0064 |
| 3T | 0.0066 | 0.0068 | 0.0069 | 0.0073 | 0.0067 | 0.0062 | 0.0070 | 0.0067 | 0.0065 | 0.0063 | 0.0077 | 0.0342 | 0.0059 | 0.0078 | 0.0064 | 0.0067 | 0.0705 | 0.0085 | 0.0065 |  | 0.0065 | 0.0071 | 0.0061 | 0.0072 | 0.0063 |
| SC | 0.0060 | 0.0072 | 0.0071 | 0.0148 | 0.0067 | 0.0063 | 0.0059 | 0.0068 | 0.0058 | 0.0073 | 0.0068 | 0.9967 | 0.0068 | 0.0185 | 0.0067 | 0.0067 | 0.0062 | 0.0069 | 0.0278 | 0.0064 |  | 0.0063 | 0.0061 | 0.0071 | 0.0075 |
| 48T | 0.0071 | 0.0069 | 0.0066 | 0.0069 | 0.0064 | 0.0065 | 0.0067 | 0.0067 | 0.0061 | 0.0058 | 0.0067 | 0.0062 | 0.0062 | 0.0899 | 0.0065 | 0.0063 | 0.0066 | 0.0065 | 0.0072 | 0.0069 | 0.0061 |  | 0.0064 | 0.0066 | 0.0285 |
| BC | 0.0049 | 0.0063 | 0.0058 | 0.0055 | 0.0068 | 0.0059 | 0.0066 | 0.0062 | 0.0117 | 0.0060 | 0.0060 | 0.0071 | 0.0059 | 0.0120 | 0.0067 | 0.0095 | 0.0065 | 0.0071 | 0.0066 | 0.0059 | 0.0067 | 0.0054 |  | 0.0061 | 0.006 |
| YP | 0.0058 | 0.0071 | 0.0067 | 0.0072 | 0.0062 | 0.0066 | 0.0063 | 0.0063 | 0.0077 | 0.0059 | 0.0061 | 0.0055 | 0.0066 | 0.0073 | 0.0063 | 0.0061 | 0.0068 | 0.0069 | 0.0070 | 0.0064 | 0.0074 | 0.0075 | 0.0067 |  | 0.0061 |
| ZP | 0.0067 | 0.0059 | 0.0064 | 0.0064 | 0.0063 | 0.0062 | 0.0062 | 0.0060 | 0.0080 | 0.0082 | 0.0452 | 0.0075 | 0.0063 | 0.0164 | 0.0065 | 0.0057 | 0.0251 | 0.0063 | 0.0064 | 0.0063 | 0.0059 | 0.0065 | 0.0077 | 0.0061 |  |
