# Supplementary material for: Population Genetic Structure of Glycyrrhiza inflata B. (Fabaceae) Is Shaped by Habitat Fragmentation, Water Resources and Biological Characteristics
Source: PLoS One. 2016 Oct 6;11(10):e0164129. doi: 10.1371/journal.pone.0164129 (PMC5053598; doi:10.1371/journal.pone.0164129)
Supplement: S3 Table — (DOC) [file pone.0164129.s003.doc]

**S3 Table.** Historical migration rates between 25 populations of *G.inflata* in China.

|  | GJJ | GYX | GGG | XXX | HS | HJ | SS | TMG | WL | 34T | KC | SY | EM | 8T | 3T | BC | 48T | YP | SC | ZP | LP | CL | MF | QM | RQ |
| --- | --- | --- | --- | --- | --- | --- | --- | --- | --- | --- | --- | --- | --- | --- | --- | --- | --- | --- | --- | --- | --- | --- | --- | --- | --- |
| GJJ |  | 0.119 | 0.098 | 0.097 | 0.154 | 0.049 | 0.132 | 0.110 | 0.141 | 0.172 | 0.059 | 0.097 | 0.121 | 0.113 | 0.103 | 0.106 | 0.133 | 0.067 | 0.172 | 0.097 | 0.136 | 0.164 | 0.089 | 0.117 | 0.097 |
| GYX | 0.168 |  | 0.135 | 0.108 | 0.055 | 0.119 | 0.171 | 0.106 | 0.117 | 0.108 | 0.112 | 0.126 | 0.171 | 0.123 | 0.147 | 0.076 | 0.077 | 0.103 | 0.095 | 0.193 | 0.142 | 0.157 | 0.076 | 0.222 | 0.138 |
| GGG | 0.088 | 0.049 |  | 0.091 | 0.100 | 0.142 | 0.094 | 0.096 | 0.091 | 0.078 | 0.089 | 0.072 | 0.055 | 0.127 | 0.099 | 0.107 | 0.091 | 0.109 | 0.080 | 0.073 | 0.065 | 0.068 | 0.138 | 0.098 | 0.136 |
| XXX | 0.046 | 0.108 | 0.121 |  | 0.125 | 0.111 | 0.092 | 0.107 | 0.095 | 0.152 | 0.071 | 0.082 | 0.072 | 0.101 | 0.129 | 0.115 | 0.115 | 0.099 | 0.130 | 0.132 | 0.161 | 0.086 | 0.073 | 0.221 | 0.125 |
| HS | 0.092 | 0.089 | 0.092 | 0.122 |  | 0.129 | 0.096 | 0.103 | 0.086 | 0.094 | 0.039 | 0.112 | 0.131 | 0.094 | 0.091 | 0.076 | 0.126 | 0.112 | 0.090 | 0.102 | 0.142 | 0.168 | 0.104 | 0.078 | 0.116 |
| HJ | 0.133 | 0.126 | 0.145 | 0.089 | 0.139 |  | 0.146 | 0.074 | 0.143 | 0.134 | 0.134 | 0.141 | 0.086 | 0.093 | 0.134 | 0.112 | 0.078 | 0.106 | 0.150 | 0.126 | 0.097 | 0.107 | 0.060 | 0.127 | 0.104 |
| SS | 0.154 | 0.134 | 0.163 | 0.127 | 0.104 | 0.125 |  | 0.189 | 0.122 | 0.075 | 0.161 | 0089 | 0.211 | 0.152 | 0.132 | 0.119 | 0.156 | 0.074 | 0.099 | 0.081 | 0.198 | 0.184 | 0.250 | 0.089 | 0.097 |
| TMG | 0.124 | 0.158 | 0.173 | 0.133 | 0.159 | 0.152 | 0.187 |  | 0.118 | 0.113 | 0.171 | 0.184 | 0.182 | 0.180 | 0.150 | 0.183 | 0.102 | 0.120 | 0.099 | 0.084 | 0.190 | 0.173 | 0.140 | 0.129 | 0.122 |
| WL | 0.077 | 0.113 | 0.073 | 0.097 | 0.095 | 0.045 | 0.068 | 0.063 |  | 0.097 | 0.056 | 0.102 | 0.081 | 0.065 | 0.072 | 0.089 | 0.048 | 0.060 | 0.079 | 0.075 | 0.034 | 0.105 | 0.071 | 0.046 | 0.115 |
| 34T | 0.204 | 0.163 | 0.124 | 0.098 | 0.081 | 0.128 | 0.117 | 0.124 | 0.145 |  | 0.091 | 0.178 | 0.112 | 0.121 | 0.117 | 0.135 | 0.119 | 0.114 | 0.147 | 0.162 | 0.110 | 0.109 | 0.112 | 0.107 | 0.096 |
| KC | 0.120 | 0.172 | 0.106 | 0.074 | 0.161 | 0.140 | 0.095 | 0.065 | 0.085 | 0.175 |  | 0.138 | 0.151 | 0.134 | 0.108 | 0.195 | 0.135 | 0.115 | 0.105 | 0.179 | 0.123 | 0.119 | 0.217 | 0.114 | 0.116 |
| SY | 0.104 | 0.117 | 0.133 | 0.033 | 0.138 | 0.065 | 0.102 | 0.152 | 0.175 | 0.089 | 0.143 |  | 0.165 | 0.122 | 0.178 | 0.101 | 0.188 | 0.149 | 0.067 | 0.128 | 0.098 | 0.146 | 0.159 | 0.051 | 0.154 |
| EM | 0.091 | 0.077 | 0.097 | 0.113 | 0.133 | 0.131 | 0.166 | 0.091 | 0.135 | 0.144 | 0.171 | 0.124 |  | 0.091 | 0.113 | 0.067 | 0.152 | 0.083 | 0.062 | 0.098 | 0.090 | 0.221 | 0.105 | 0.127 | 0.081 |
| 8T | 0.050 | 0.099 | 0.096 | 0.052 | 0.093 | 0.141 | 0.139 | 0.083 | 0.121 | 0.033 | 0.092 | 0.102 | 0.047 |  | 0.112 | 0.092 | 0.084 | 0.092 | 0.090 | 0.095 | 0.138 | 0.086 | 0.106 | 0.142 | 0.085 |
| 3T | 0.187 | 0.066 | 0.138 | 0.131 | 0.179 | 0.186 | 0.105 | 0.121 | 0.104 | 0.123 | 0.104 | 0.068 | 0.063 | 0.122 |  | 0.134 | 0.113 | 0.122 | 0.149 | 0.087 | 0.104 | 0.079 | 0.123 | 0.139 | 0.165 |
| BC | 0.108 | 0.088 | 0.059 | 0.073 | 0.108 | 0.157 | 0.069 | 0.101 | 0.054 | 0.196 | 0.078 | 0.106 | 0.135 | 0.080 | 0.137 |  | 0.071 | 0.133 | 0.094 | 0.178 | 0.084 | 0.078 | 0.182 | 0.076 | 0.119 |
| 48T | 0.079 | 0.166 | 0.144 | 0.121 | 0.120 | 0.075 | 0.111 | 0.143 | 0.080 | 0.155 | 0.137 | 0.104 | 0.144 | 0.068 | 0.127 | 0.124 |  | 0.108 | 0.134 | 0.141 | 0.128 | 0.139 | 0.089 | 0.095 | 0.085 |
| YP | 0.137 | 0.156 | 0.110 | 0.067 | 0.103 | 0.123 | 0.159 | 0.127 | 0.112 | 0.123 | 0.094 | 0.119 | 0.096 | 0.129 | 0.123 | 0.135 | 0.122 |  | 0.097 | 0.132 | 0.149 | 0.153 | 0.196 | 0.099 | 0.098 |
| SC | 0.124 | 0.137 | 0.143 | 0.128 | 0.164 | 0.157 | 0.095 | 0.201 | 0.091 | 0.078 | 0.126 | 0.089 | 0.073 | 0.092 | 0.123 | 0.118 | 0.095 | 0.218 |  | 0.094 | 0.084 | 0.164 | 0.068 | 0.097 | 0.129 |
| ZP | 0.119 | 0.123 | 0.079 | 0.081 | 0.121 | 0.061 | 0.088 | 0.085 | 0.069 | 0.089 | 0.080 | 0.123 | 0.113 | 0.070 | 0.073 | 0.137 | 0.114 | 0.105 | 0.075 |  | 0.077 | 0。071 | 0.083 | 0.083 | 0.081 |
| LP | 0.086 | 0.096 | 0.110 | 0.098 | 0.066 | 0.082 | 0.051 | 0.082 | 0.134 | 0.136 | 0.161 | 0.099 | 0.109 | 0.119 | 0.119 | 0.099 | 0.093 | 0.121 | 0.098 | 0.151 |  | 0.136 | 0.077 | 0.135 | 0.132 |
| CL | 0.178 | 0.078 | 0.133 | 0.137 | 0.091 | 0.085 | 0.071 | 0.164 | 0.114 | 0.106 | 0.115 | 0.136 | 0.077 | 0.076 | 0.066 | 0.128 | 0.121 | 0.069 | 0.202 | 0.076 | 0.072 |  | 0.058 | 0.124 | 0.072 |
| MF | 0.091 | 0.110 | 0.086 | 0.150 | 0.060 | 0.139 | 0.071 | 0.231 | 0.088 | 0.115 | 0.154 | 0.104 | 0.095 | 0.113 | 0.088 | 0.131 | 0.107 | 0.132 | 0.072 | 0.113 | 0.113 | 0.131 |  | 0.072 | 0.093 |
| QM | 0.106 | 0.123 | 0.146 | 0.096 | 0.119 | 0.083 | 0.183 | 0.081 | 0.112 | 0.081 | 0.135 | 0.173 | 0.086 | 0.091 | 0.076 | 0.117 | 0.090 | 0.089 | 0.082 | 0.051 | 0.111 | 0.158 | 0.117 |  | 0.131 |
| RQ | 0.145 | 0.089 | 0.107 | 0.075 | 0.111 | 0.096 | 0.079 | 0.081 | 0.073 | 0.057 | 0.080 | 0.094 | 0.080 | 0.077 | 0.103 | 0.182 | 0.080 | 0.125 | 0.157 | 0.052 | 0.140 | 0.085 | 0.092 | 0.084 |  |
