# Supplementary material for: Population Genetic Structure of Glycyrrhiza inflata B. (Fabaceae) Is Shaped by Habitat Fragmentation, Water Resources and Biological Characteristics
Source: PLoS One. 2016 Oct 6;11(10):e0164129. doi: 10.1371/journal.pone.0164129 (PMC5053598; doi:10.1371/journal.pone.0164129)
Supplement: S4 Table — (DOC) [file pone.0164129.s004.doc]

**S4 Table.** The sample IDs of the ten best core collections

| Samples ID | F01 core | F02 core | F03 core | F04 core | F05 core | F06 core | F07 core | F08 core | F09 core | F10 core |
| --- | --- | --- | --- | --- | --- | --- | --- | --- | --- | --- |
| 1 | GJJ5 | GYXB1 | GYXB1 | GYXB1 | GYXB1 | GYXB1 | GGGB11 | GJJ7 | GJJ8 | GYXB1 |
| 2 | GYXB1 | GYXB12 | GYXB5 | GYXB12 | GYXB12 | GYXB12 | GYXB1 | GYXB1 | GYXB1 | GYXB12 |
| 3 | GYXB7 | 34T8 | GYXB12 | 34T11 | 34T11 | 34T3 | GYXB12 | GYXB12 | GYXB12 | 34T8 |
| 4 | GYXB12 | 34T9 | 34T9 | 34T12 | 34T12 | 34T9 | 34T9 | 34T8 | 34T9 | 34T9 |
| 5 | 34T9 | 34T11 | 34T11 | 34T14 | 34T14 | 34T10 | 34T11 | 34T9 | 34T11 | 34T11 |
| 6 | 34T11 | 34T12 | 34T12 | 34T15 | 34T15 | 34T11 | 34T12 | 34T11 | 34T12 | 34T12 |
| 7 | 34T12 | 3T4 | 34T14 | 34T9 | 34T9 | 48T2 | 34T14 | 34T12 | 48T2 | 48T2 |
| 8 | 34T14 | 3T11 | 48T2 | 3T2 | 3T8 | 48T12 | 48T2 | 48T2 | 48T11 | 48T12 |
| 9 | 3T6 | 48T2 | 48T12 | 3T6 | 3T12 | 48T14 | 48T12 | 48T12 | 48T12 | 48T14 |
| 10 | 48T2 | 48T12 | 48T16 | 48T2 | 48T12 | 48T16 | 48T16 | 48T14 | 48T14 | 8T12 |
| 11 | 48T12 | 48T14 | 8T12 | 48T12 | 48T16 | 8T12 | 8T12 | 48T16 | 8T1 | BC11 |
| 12 | 48T16 | 48T16 | BC3 | 48T16 | 48T2 | BC10 | BC11 | 8T12 | 8T12 | BC15 |
| 13 | 8T12 | 8T12 | BC11 | 8T12 | 8T12 | BC11 | BC18 | BC11 | BC2 | CL2 |
| 14 | BC4 | BC8 | CL2 | BC11 | BC11 | CL2 | CL2 | BC17 | BC16 | CL4 |
| 15 | BC11 | BC11 | CL4 | BC13 | BC14 | CL4 | CL4 | CL2 | CL2 | CL7 |
| 16 | BC16 | CL2 | CL16 | CL2 | CL2 | CL6 | EM6 | CL4 | CL4 | EM5 |
| 17 | CL2 | CL3 | EM7 | CL3 | CL4 | CL7 | EM9 | CL7 | EM6 | EM9 |
| 18 | CL4 | CL7 | EM9 | EM6 | CL7 | EM3 | EM15 | EM6 | EM9 | EM15 |
| 19 | CL11 | EM3 | EM15 | EM9 | EM7 | EM9 | LB2 | EM8 | EM15 | LB2 |
| 20 | EM9 | EM9 | LB2 | EM15 | EM9 | EM15 | LB4 | EM9 | LB2 | LB4 |
| 21 | EM12 | EM15 | LB4 | LB2 | EM15 | LB2 | 124Nb3 | EM15 | LB4 | XT2 |
| 22 | EM15 | LB2 | XT2 | LB5 | LB2 | LB5 | 124Nb4 | LB2 | LB7 | 124Nb5 |
| 23 | LB2 | LB4 | XT9 | 124Nb5 | LB4 | 124Nb5 | 124Nb5 | LB4 | 124Nb5 | KC8 |
| 24 | LB4 | 124Nb1 | 124Nb5 | XT2 | 124Nb5 | 124Nb10 | 124Nb8 | XT2 | 124Nb10 | KC9 |
| 25 | XT2 | 124Nb2 | KC9 | KC10 | XT2 | XT2 | XT2 | 124Nb5 | XT2 | LP8 |
| 26 | 124Nb5 | 124Nb5 | KC10 | LP13 | KC8 | KC9 | KC9 | KC9 | KC9 | MF6 |
| 27 | KC10 | XT2 | LP14 | MF6 | KC9 | KC14 | KC10 | KC10 | KC14 | MF15 |
| 28 | LP14 | KC9 | MF2 | MF15 | MF6 | MF6 | LP14 | LP8 | LP14 | QM3 |
| 29 | MF6 | KC14 | MF6 | QM3 | MF8 | MF15 | MF6 | LP14 | MF6 | QM5 |
| 30 | MF15 | MF1 | MF15 | QM5 | MF15 | QM4 | MF15 | MF6 | MF15 | QM11 |
| 31 | QM5 | MF6 | QM5 | QM12 | QM4 | QM5 | QM5 | MF15 | QM5 | QM12 |
| 32 | QM12 | MF15 | QM8 | RQ6 | QM5 | QM12 | QM9 | QM5 | QM12 | RQ5 |
| 33 | QM14 | QM3 | QM9 | RQ8 | QM12 | RQ2 | QM12 | QM12 | QM14 | RQ9 |
| 34 | RQ5 | QM5 | QM12 | RQ15 | RQ5 | RQ15 | RQ5 | RQ3 | RQ3 | RQ15 |
| 35 | RQ15 | QM12 | RQ5 | SC1 | RQ11 | SC1 | RQ8 | RQ13 | RQ15 | SC1 |
| 36 | SC1 | RQ7 | RQ7 | SC5 | SC1 | SC5 | RQ15 | SC1 | SC1 | SC11 |
| 37 | SC5 | RQ13 | RQ15 | SC11 | SC7 | SC11 | SC1 | SC11 | SC11 | SC14 |
| 38 | SC11 | SC1 | SC1 | DKE3 | SC11 | DKE3 | SC10 | SC16 | SC16 | SC16 |
| 39 | SC15 | SC11 | SC11 | DKE7 | SC16 | DKE7 | SC11 | DKE1 | DKE3 | DKE3 |
| 40 | DKE6 | SC16 | SC16 | LMQ4 | DKE6 | LMQ4 | SC16 | LMQ3 | DKE7 | DKE7 |
| 41 | DKE7 | DKE3 | LMQ12 | LMQ8 | DKE7 | LMQ8 | DKE3 | LMQ6 | LMQ4 | MQ4 |
| 42 | LMQ4 | DKE7 | DKE3 | LMQ16 | LMQ1 | LMQ16 | DKE7 | LMQ8 | LMQ8 | MQ8 |
| 43 | LMQ12 | LMQ4 | DKE7 | SY2 | LMQ4 | SY2 | LMQ4 | DKE7 | LMQ16 | MQ16 |
| 44 | LMQ16 | LMQ8 | LMQ4 | SY4 | LMQ8 | SY4 | LMQ8 | LMQ4 | SY2 | SY2 |
| 45 | SY2 | LMQ16 | LMQ16 | TMG2 | LMQ16 | SY5 | LMQ16 | LMQ16 | SY4 | SY4 |
| 46 | SY4 | SY2 | SY2 | TMG3 | SY2 | SY9 | SY2 | SY1 | TMG2 | TMG2 |
| 47 | TMG2 | SY4 | SY4 | TMG5 | SY4 | SY12 | SY4 | SY2 | TMG3 | TMG3 |
| 48 | TMG3 | TMG2 | TMG2 | TMG18 | TMG2 | TMG2 | TMG2 | SY4 | TMG5 | TMG5 |
| 49 | TMG5 | TMG3 | TMG3 | 31T5 | TMG3 | TMG3 | TMG3 | TMG2 | 31T10 | TMG18 |
| 50 | 31T8 | TMG5 | TMG5 | 31T9 | 31T8 | TMG5 | TMG5 | TMG3 | XXX6 | 31T6 |
| 51 | 31T10 | 31T9 | 31T11 | 31T10 | TLM4 | TLM14 | TLM7 | TMG5 | XXX10 | TLM14 |
| 52 | TLM10 | XXX5 | XXX13 | XXX10 | XXX10 | 31T9 | 31T10 | TLM7 | XXX17 | XXX6 |
| 53 | XXX6 | XXX10 | XXX16 | XXX16 | XXX17 | XXX6 | XXX6 | 31T9 | XXX18 | XXX14 |
| 54 | XXX14 | XXX14 | XXX17 | XXX17 | XXX18 | XXX14 | XXX14 | XXX10 | XXX22 | XXX18 |
| 55 | XXX18 | XXX18 | XXX18 | XXX18 | XXX22 | XXX18 | XXX18 | XXX14 | YP13 | YP9 |
| 56 | ZP12 | XXX23 | ZP12 | ZP14 | ZP12 | YP9 | ZP12 | XXX18 | ZP12 | ZP14 |
| 57 | ZP15 | ZP12 | ZP14 | ZP15 | ZP14 | ZP12 | ZP15 | ZP12 | ZP14 | ZP18 |
