# Supplementary material for: Population Genetic Structure of Glycyrrhiza inflata B. (Fabaceae) Is Shaped by Habitat Fragmentation, Water Resources and Biological Characteristics
Source: PLoS One. 2016 Oct 6;11(10):e0164129. doi: 10.1371/journal.pone.0164129 (PMC5053598; doi:10.1371/journal.pone.0164129)
Supplement: S5 Table — (DOC) [file pone.0164129.s005.doc]

**S5 Table.** The number of core individuals in every population

| Population | F01 core | F02 core | F03 core | F04 core | F05 core | F06 core | F07 core | F08 core | F09 core | F10 core | *N* | *K* |
| --- | --- | --- | --- | --- | --- | --- | --- | --- | --- | --- | --- | --- |
| GJJ | 1 | 0 | 0 | 0 | 0 | 0 | 0 | 1 | 1 | 0 | 3 | 30.00 |
| GYX | 3 | 2 | 3 | 2 | 2 | 2 | 2 | 2 | 2 | 2 | 4 | 23.53 |
| GGG | 0 | 0 | 0 | 0 | 0 | 0 | 1 | 0 | 0 | 0 | 1 | 5.26 |
| XXX | 3 | 5 | 4 | 4 | 4 | 3 | 3 | 3 | 5 | 3 | 10 | 58.82 |
| SS | 5 | 5 | 5 | 5 | 6 | 5 | 5 | 7 | 7 | 7 | 11 | 73.33 |
| HJ | 2 | 2 | 2 | 2 | 2 | 2 | 2 | 2 | 3 | 2 | 9 | 52.94 |
| HS | 2 | 4 | 3 | 2 | 2 | 3 | 5 | 2 | 3 | 2 | 9 | 45.00 |
| 34T | 4 | 4 | 4 | 5 | 5 | 4 | 4 | 4 | 3 | 4 | 8 | 53.33 |
| TMG | 3 | 3 | 3 | 4 | 2 | 3 | 3 | 3 | 3 | 4 | 4 | 33.33 |
| KC | 1 | 2 | 2 | 1 | 2 | 2 | 2 | 2 | 2 | 2 | 4 | 26.67 |
| RQ | 2 | 2 | 3 | 3 | 2 | 2 | 3 | 2 | 2 | 3 | 10 | 66.67 |
| QM | 3 | 3 | 4 | 3 | 3 | 3 | 3 | 2 | 3 | 4 | 8 | 53.33 |
| LP | 1 | 0 | 1 | 1 | 0 | 0 | 1 | 2 | 1 | 1 | 3 | 20.00 |
| CL | 3 | 3 | 3 | 2 | 3 | 4 | 2 | 3 | 2 | 3 | 7 | 43.75 |
| MF | 2 | 3 | 3 | 2 | 3 | 2 | 2 | 2 | 2 | 2 | 3 | 20.00 |
| WL | 3 | 1 | 1 | 3 | 2 | 2 | 2 | 2 | 1 | 2 | 10 | 47.62 |
| SY | 2 | 2 | 2 | 2 | 2 | 5 | 2 | 3 | 2 | 2 | 6 | 42.86 |
| EM | 3 | 3 | 3 | 3 | 3 | 3 | 3 | 4 | 3 | 3 | 8 | 53.33 |
| 8T | 1 | 1 | 1 | 1 | 1 | 1 | 1 | 1 | 2 | 1 | 2 | 12.50 |
| 3T | 1 | 2 | 0 | 2 | 2 | 0 | 0 | 0 | 0 | 0 | 6 | 40.00 |
| SC | 4 | 3 | 3 | 3 | 4 | 3 | 4 | 3 | 3 | 4 | 8 | 50.00 |
| 48T | 3 | 4 | 3 | 3 | 3 | 4 | 3 | 4 | 4 | 3 | 5 | 31.25 |
| BC | 3 | 2 | 2 | 2 | 2 | 2 | 2 | 2 | 2 | 2 | 12 | 66.67 |
| YP | 0 | 0 | 0 | 0 | 0 | 1 | 0 | 0 | 1 | 1 | 2 | 13.33 |
| ZP | 2 | 1 | 2 | 2 | 2 | 1 | 2 | 1 | 2 | 2 | 4 | 22.22 |
|  | 57 | 57 | 57 | 57 | 57 | 57 | 57 | 57 | 57 | 57 | 175 |  |

Notes: *N*: The number of core individuals in the population. *K*: The percentage of core individuals in the population.
