# Supplementary material for: Population Genetic Structure of Glycyrrhiza inflata B. (Fabaceae) Is Shaped by Habitat Fragmentation, Water Resources and Biological Characteristics
Source: PLoS One. 2016 Oct 6;11(10):e0164129. doi: 10.1371/journal.pone.0164129 (PMC5053598; doi:10.1371/journal.pone.0164129)
Supplement: S6 Table — (DOC) [file pone.0164129.s006.doc]

**S6 Table.** The historical and contemporary migration rates of LP and neighbour populations from two clusters

|  | **m_h_** | | | | | **m_c_** | | | | |
| --- | --- | --- | --- | --- | --- | --- | --- | --- | --- | --- |
|  | LP | SC | 48T | BC | ZP | LP | SC | 48T | BC | ZP |
| LP |  | 0.0978 | 0.0926 | 0.0985 | 0.1507 |  | 0.0063 | 0.0075 | 0.0066 | 0.0054 |
| SC | 0.0841 |  | 0.0946 | 0.1181 | 0.0944 | 0.0068 |  | 0.0063 | 0.0061 | 0.0075 |
| 48T | 0.1282 | 0.1336 |  | 0.1242 | 0.1409 | 0.0062 | 0.0061 |  | 0.0064 | 0.0285 |
| BC | 0.0839 | 0.0941 | 0.0707 |  | 0.1783 | 0.0059 | 0.0067 | 0.0054 |  | 0.0060 |
| ZP | 0.0772 | 0.0749 | 0.1139 | 0.1373 |  | 0.0063 | 0.0059 | 0.0065 | 0.0077 |  |
| Mean(→X) | 0.1100 | 0.0979 | 0.1318 | 0.1068 | 0.1008 | 0.0065 | 0.0049 | 0.0102 | 0.0045 | 0.0051 |
| Mean(X→) | 0.0934 | 0.1002 | 0.0930 | 0.1196 | 0.1411 | 0.0063 | 0.0063 | 0.0064 | 0.0067 | 0.0119 |
| Mean | 0.1017 | 0.0990 | 0.1124 | 0.1132 | 0.1210 | 0.0063 | 0.0056 | 0.0083 | 0.0056 | 0.0084 |

Notes: X: The populations in row
