# Supplementary material for: Population Genetic Structure of Glycyrrhiza inflata B. (Fabaceae) Is Shaped by Habitat Fragmentation, Water Resources and Biological Characteristics
Source: PLoS One. 2016 Oct 6;11(10):e0164129. doi: 10.1371/journal.pone.0164129 (PMC5053598; doi:10.1371/journal.pone.0164129)
Supplement: S7 Table — (DOC) [file pone.0164129.s007.doc]

**S7 Table** The historical and contemporary migration rates of HJ, HS, TMG and neighbour populations from two clusters

|  | **m_h_** | | | | | | **m_c_** | | | | | |
| --- | --- | --- | --- | --- | --- | --- | --- | --- | --- | --- | --- | --- |
|  | SS | HJ | HS | TMG | KC | SY | SS | HJ | HS | TMG | KC | SY |
| SS |  | 0.1250 | 0.1039 | 0.1886 | 0.1613 | 0.0892 |  | 0.0074 | 0.0080 | 0.0076 | 0.0080 | 0.0086 |
| HJ | 0.1459 |  | 0.1389 | 0.0735 | 0.1340 | 0.1411 | 0.0079 |  | 0.0073 | 0.0740 | 0.0071 | 0.0079 |
| HS | 0.0955 | 0.1298 |  | 0.1031 | 0.0396 | 0.1115 | 0.0071 | 0.0071 |  | 0.0488 | 0.0073 | 0.0106 |
| TMG | 0.1873 | 0.1525 | 0.1587 |  | 0.1706 | 0.1836 | 0.0070 | 0.0065 | 0.0072 |  | 0.0087 | 0.0081 |
| KC | 0.0953 | 0.1403 | 0.1609 | 0.0645 |  | 0.1384 | 0.0069 | 0.0070 | 0.0071 | 0.0121 |  | 0.0099 |
| SY | 0.1017 | 0.0655 | 0.1380 | 0.1523 | 0.1434 |  | 0.0064 | 0.0073 | 0.0065 | 0.0065 | 0.0067 |  |
| Mean(→X) | 0.1335 | 0.1266 | 0.0959 | 0.1705 | 0.1198 | 0.1202 | 0.0078 | 0.0208 | 0.0162 | 0.0075 | 0.0086 | 0.0067 |
| Mean(X→) | 0.1251 | 0.1226 | 0.1401 | 0.1164 | 0.1298 | 0.1327 | 0.0070 | 0.0070 | 0.0072 | 0.0298 | 0.0075 | 0.0090 |
| Mean | 0.1293 | 0.1246 | 0.1180 | 0.1434 | 0.1248 | 0.1264 | 0.0074 | 0.0139 | 0.0117 | 0.0186 | 0.0087 | 0.0078 |

Notes: X: The populations in row
